# Pattern dystrophies in patients treated with deferoxamine: report of two cases and review of the literature

**DOI:** 10.1186/s12886-018-0911-2

**Published:** 2018-09-12

**Authors:** Constantine D. Georgakopoulos, Foteini Tsapardoni, Elli V. Kostopoulou, Olga E. Makri

**Affiliations:** Department of Ophthalmology, Medical School, University of Patras, 265 04 Patras, Greece

**Keywords:** Deferoxamine, Pattern dystrophy, Thalassemia, Drug toxicity

## Abstract

**Background:**

Deferoxamine (DFO) is one of the most commonly used chelation treatments for transfusional hemosiderosis. Pattern dystrophies constitute a distinct entity of retinal disorders that has been occasionally identified in association with deferoxamine.

**Case presentation:**

We report two cases of bilateral macular pattern dystrophy in transfusion dependent patients undergoing chronic chelation therapy with deferoxamine due to thalassemias. Our patients were evaluated with multimodal imaging and the results are presented. Both patients had normal cone and rod responses in the full-field electroretinogram and continued the prescribed chelation therapy, after hematology consult. The patients were followed up every 3 months for 2 and 4 years respectively for possible deterioration. Their best corrected visual acuity remained stable with no anatomic change on Optical Coherence Tomography findings.

**Conclusion:**

Multimodal imaging of our patients allowed a better evaluation and possibly earlier detection of the DFO-related changes. Screening and close follow up of patients under chronic chelating therapy is important in order to promptly diagnose and manage possible toxicity either with discontinuation of the offending agent or dose modification.

## Background

Thalassemias are a group of inherited blood disorders characterized by imbalance in hemoglobulin production. Patients with thalassemia require frequent blood transfusions to survive, resulting in iron overload that necessitates chelation therapy [1]. The most commonly used iron chelators are deferoxamine (DFO), deferiprone and deferasirox. Chronic use of chelating agents, especially DFO, may cause ocular toxicity like pigmentary retinopathy or optic neuropathy presenting with color vision abnormalities, night blindness and visual fields defects [2]. Early signs indicative of DFO-induced toxicity are opacification and loss of transparency of the retinal pigment epithelium (RPE) and outer retina, which precede RPE mottling and may involve the papillomacular, peripapillar or paramacular area [3].

We present two patients undergoing chronic chelation therapy due to thalassemias with bilateral macular pattern dystrophy studied through multimodal imaging and we review the existing literature.

## Case presentation

### Case 1

Α 54 year old Caucasian woman of Greek ancestry presented to the Retina Service of our Department complaining of metamophopsia and reduced visual acuity in both eyes, gradually worsening over the past four months. Her past medical history revealed that she suffers from beta thalassemia intermedia for which she receives blood transfusion biweekly and chelation therapy for the past 14 years. Her chelation therapy consists of DFO (50 mg/kg 5 times a week by an 8-hourly subcutaneous infusion), as well as deferiprone (50 mg/kg, per os, daily). Ferritin level was within normal limits. The remainder of her medical history included hypothyroidism treated with levothyroxine. No history of color and peripheral vision changes nor hearing impairment was reported. Her past ocular history was unremarkable.

**Fig. 1:**
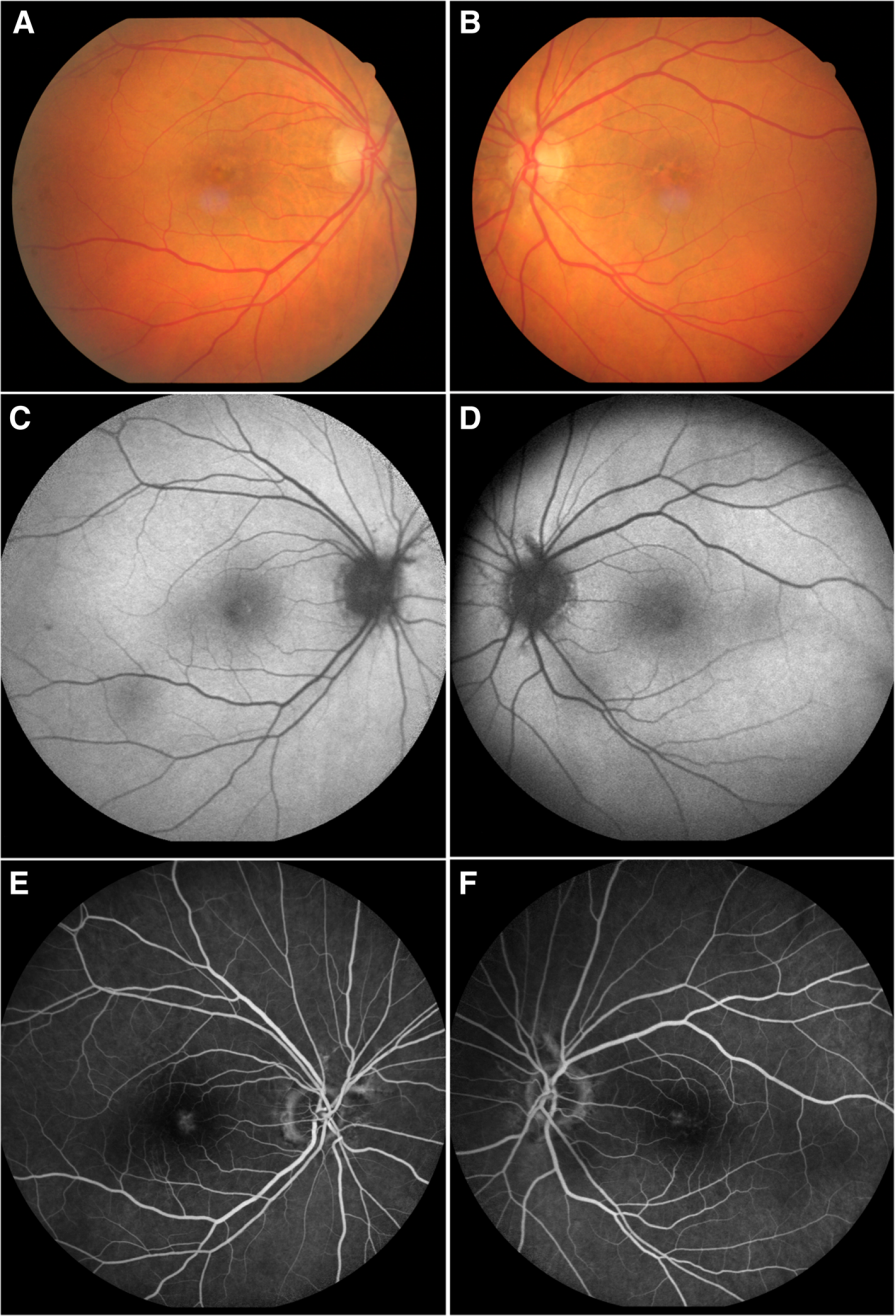
Adult onsel vitelliform macular dystrophy in a 54 year old patient (Patient 1) receiving deferoxamine chelation therapy for transfusional iron overload due to beta thalassemia intermedia. Fundus photography demonstrates the yellowish lesions with central and parafoveal hyperpigmentation in both maculas as well as angioid streaks arising from both optic discs (**a**, **b**). Mild stippled hyperautofluorescence in the borders of the macular lesions are shown in fundus autofluorescence (**c**, **d**). Fluorescein angiography reveals a subtle ring of hyperfluorescence with late staining surrounding the hypofluorescent lesion (**e**, **f**)

On the initial examination, best corrected visual acuity (BCVA) was 20/25 in the right eye (OD) and 20/22 in the left eye (OS). Slit lamp biomicroscopy revealed no pathology from the anterior segment and intraocular pressure (IOP) measured by applanation tonometry was 14 mmHg in both eyes (OU). Dilated fundus examination showed a yellowish roundish macular lesion surrounded by RPE changes, as well as angioid streaks emanating from the optic disc sparing the macula in OU (Fig. 1a, b). The retinal vasculature appeared normal.

**Fig. 2:**
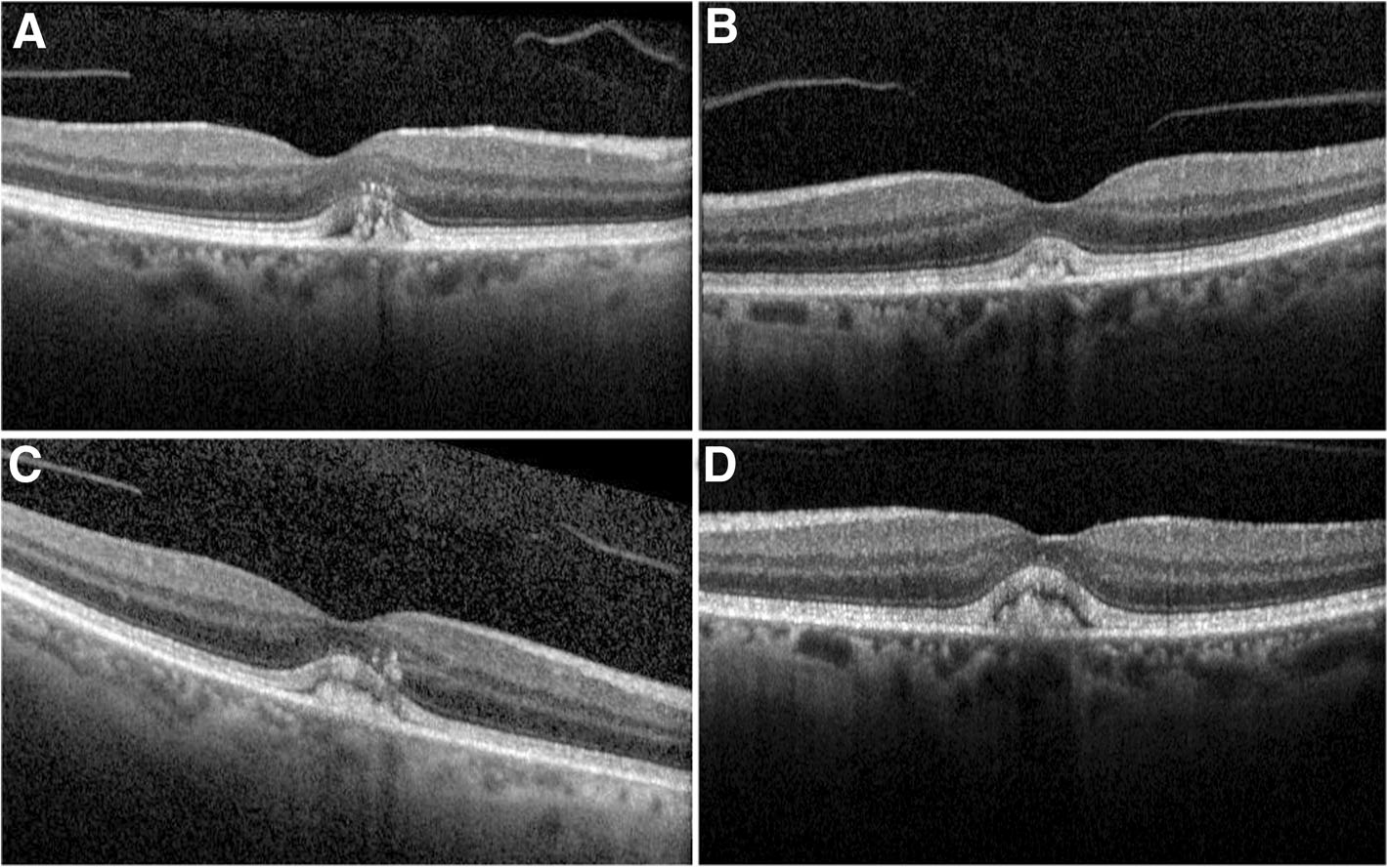
Optical coherence tomography of Patient 1 at the time of diagnosis shows material accumulation in both eyes causing a dome shaped subfoveal lesion situated above the RPE (**a**, **b**). After 2 years follow up OCT of the right eye remains unchanged with a slight increase of the subfoveal debris in the left eye (**c**, **d**)

**Fig. 3:**
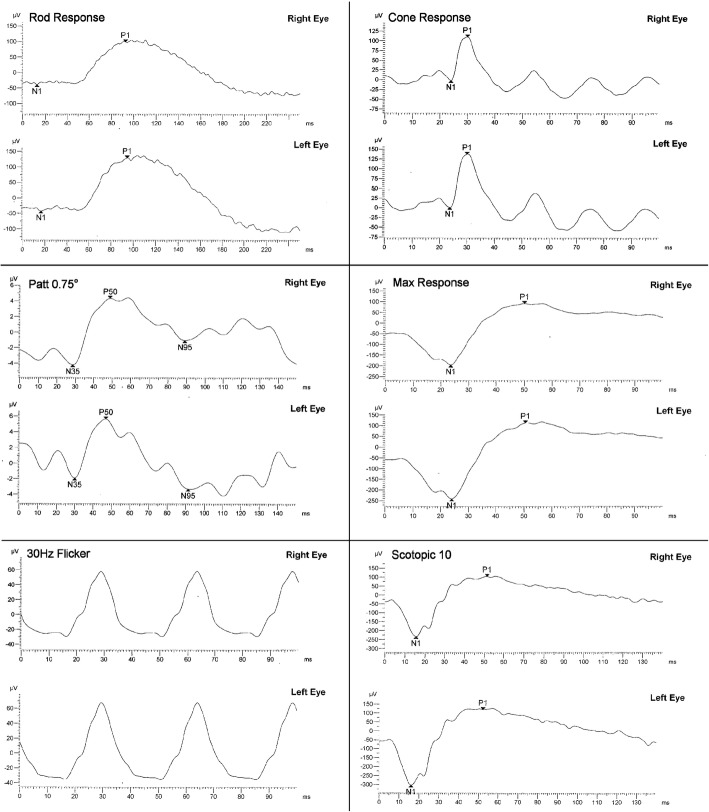
Pattern ERG and a full-field ERG of Patient 1. Pattern ERG responses showed normal P50 component bilaterally (8,5 μV-OD, 7,6 μV-OS)reflecting normalfunction of macular photoreceptors. Additionally normal cone and rod system responses were recorded on full field ERG reflecting normal overall scotopic and photopic function, bilaterally (cone system 111.8 μVand 136,3 μV, rod system 138,2 μV and 171,5 μV, OD and OS respectively)

Spectral-Domain Optical Coherence Tomography (SD-OCT) revealed a subfoveal hyperreflective deposit above the level of the RPE corresponding to the lesion observed fundoscopically (Fig. 2a, b) while mild stippling was noticed in the macular area in fundus autofluorescence (FAF) (Fig. 1c, d). Finally, fluoroscein angiography revealed staining of the macular lesion in the late phases of the angiogram (Fig. 1e, f). To evaluate possible DFO retinopathy in this patient, electrophysiology testing, more specifically, full-field electroretinogram (ffERG) and pattern electroretinogram (PERG), was performed. Amplitudes and implicit times for each major waveform component were included. The responses were taken according to the International Society for Clinical Electrophysiology of Vision (ISCEV) [4]. The ERG revealed no abnormal rode and cone responses OU (Fig. 3). Visual field examination, performed by automated perimetry (Humphrey 30–2 SITA-standard, Carl Zeiss Meditec, Dublin, CA) was also normal.

Taking into consideration the normal ffERG and after consultation with her hematologist, it was decided that the patient would remain on the prescribed chelation therapy, due to the severity of her systemic condition. The patient was followed up every 3 months in our department with SD-OCT for possible deterioration. Her BCVA remained stable for the next 2 years, with no anatomic change on OCT findings (Fig. 2c, d).

### Case 2

**Fig. 4:**
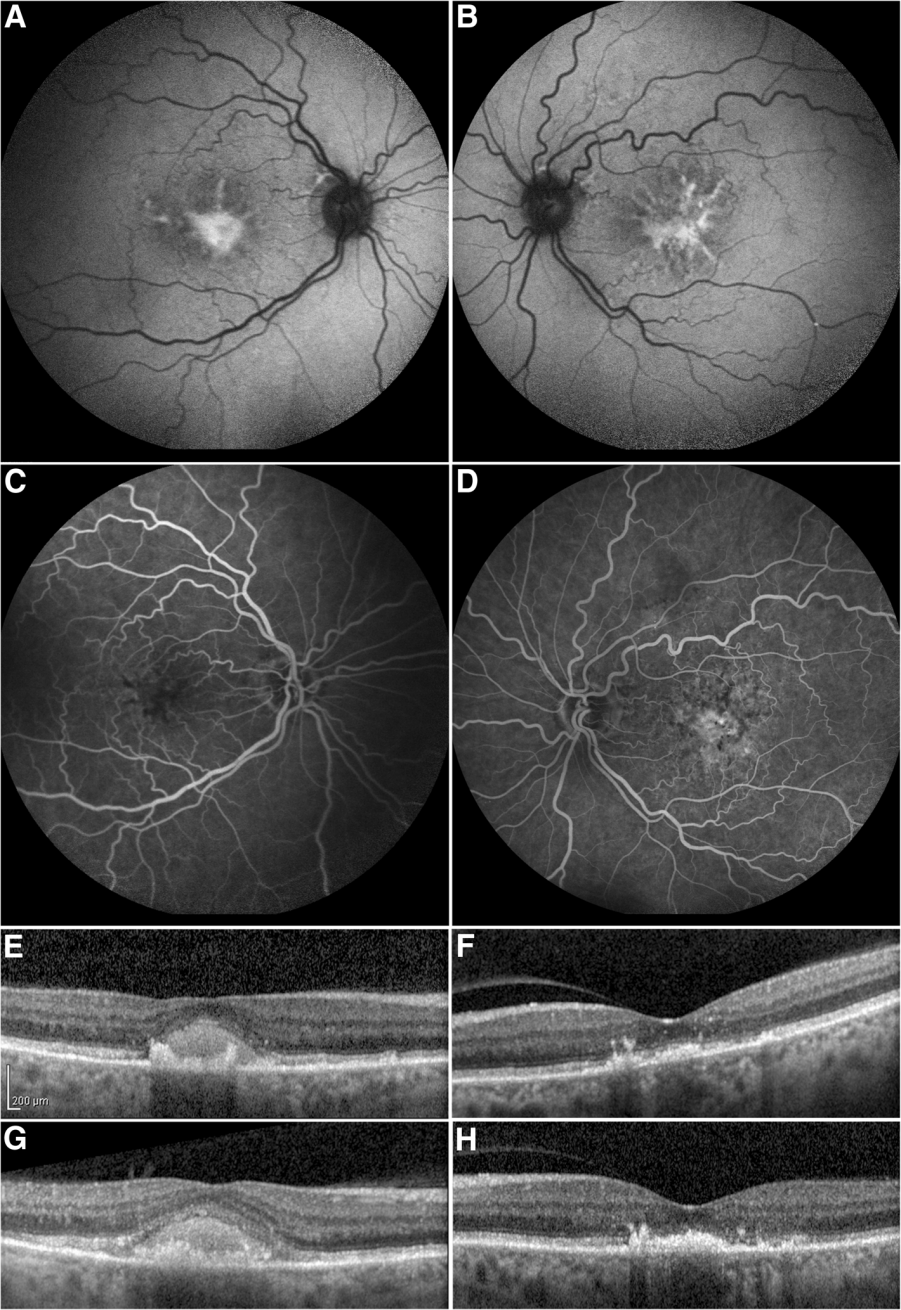
Butterfly shaped macular dystrophy in a transfusion dependent 63 year old patient receiving deferoxamine chelation therapy due to sickle beta thalassemia (Patient 2). Fundus autofluorescence shows hyperautofluorescence in a branching pattern with some areas of reduced autofluorescence corresponding to the pigment clumping seen in fundoscopy OU (**a**, **b**). Fluoroscein angiography reveals central hypofluorescence with adjacent areas of focal hyperfluorescence OU, that persists as staining without leakage in the late frames (**c**, **d**) Optical coherence tomography at the time of diagnosis showing hyperreflective material in the outer retinal layers, located at the level and above the RPE in both maculas (**e**, **f**). There is evident disruption of the ellipsoid zone by the amorphous material in the left eye. After 4 years, follow up OCT remains unchanged OS while there is a slight increase of the subfoveal debris in OD. (**g**, **h**)

A 63 year old Caucasian woman of Greek ancestry presented to our Retina Service complaining of decreased central vision OU. She had a medical history of sickle beta thalassemia for which she was receiving regular blood transfusions. She was also receiving iron-chelation treatment with DFO (50 mg/kg 5 times a week by an 8-hourly subcutaneous infusion), for the past 25 years. The rest of her medical and her ocular history were unremarkable. The patient did not report any peripheral, night or color vision problems. There was no history of hearing loss. Her BCVA was 20/50 OU. Slit lamp biomicroscopy was normal and IOP was 15 mmHg in OD and 13 mmHg in OS. Dilated fundus examination revealed the presence of a yellow-brown macular lesion OU. The lesion consisted of yellow pigment lines expanding to the perimacular area in a tri-radiating pattern surrounded by areas of granular hyper-pigmented brown material. Fundus autofluorescence revealed hyperfluorescent areas in a butterfly shaped pattern corresponding to the pigment clumping areas seen in fundoscopy OU (Fig. 4a, b). Fluoroscein angiography demonstrated a large hypofluorescent, butterfly-shaped macular lesion surrounded by areas of focal hyperfluorescence OU (Fig. 4c, d). Spectral Domain-OCT demonstrated a subfoveal hyperreflective lesion at the level and above the RPE OU (Fig. 4e, f). Electroretinogram showed no abnormal cone and rod responses. Humphrey visual field perimetry using 30–2 SITA-Standard algorithm was within normal limits.

Haematologists were consulted regarding DFO discontinuation but they decided not to make any change in chelation therapy since the risks pertaining to the patient’s underlying disease outweighed the risk of possible visual loss. Therefore, it was decided to follow-up our patient closely every three months. During a 4 years follow-up period BCVA remained stable without any signs of anatomic change as it was demonstrated with repeated OCT examinations (Fig. 4g, h).

## Discussion and conclusions

Beta-thalassemia is an autosomal recessive hemoglobinopathy resulting in anemia due to defective beta-chain globin production leading to impaired production of Hb A. Beta-thalassemia major (β-TM) is associated with diminished b-globin production, while beta-thalassemia intermedia (β-TI) is characterized by some degree of b-globin chain production. Patients with β-TI, however, may also need regular blood transfusions. Sickle beta thalassemia is a particular form of sickle cell disease in which an allele for S hemoglobulin and an allele for β-TM coexist. These patients may also require chronic blood transfusions depending on the severity of their anemia [5].

A number of ocular abnormalities have been described in patients with β-TM some of which are attributable to the natural course of the disease and the impact of the anemia on ocular tissues, while others are associated with the chronic chelation treatment [6–9]. More precisely, retinal changes present in β-TM are divided in two categories: pseudoxanthoma elasticum (PXE)-like changes that include angioid streaks, peau d’orange like fundus and optic nerve head drusen [10] and non-PXE-like changes such as increased venous tortuosity [11]. Furthermore, in a study of 255 patients with β-TM or β-TI, Barteselli et al. found a 7.5% incidence of pattern dystrophy-like changes [11].

Concerning sickle thalassemia, Aesopos et al. reported a 10% incidence of angioid streaks in a group of 58 cases [12]. Fanny et al., studied 18 patients suffering from sickle beta thalassemia and found that 13 of the patients had sickle cell retinopathy with 3 of them having proliferative disease. It appears that retinopathy in sickle thalassemia shares similar characteristics with sickle cell retinopathy [13].

Due to chronic repeated blood transfusions, these patients tend to accumulate iron in various organs such as liver, spleen, myocardium and eyes [14]. In order to prevent iron overload and possible iron-induced toxicity, chelating agents responsible for binding and excreting iron excess are administered. [1] Deferoxamine is a widely used iron-chelator available for both intravenous and subcutaneous administration. Deferoxamine related ocular toxicity involves a wide spectrum of ocular abnormalities such as nyctalopia, colour perception anomalies, visual field disturbances, cataract formation, optic neuropathy and pigmentary retinopathy [3]. Deferiprone, which is an alternative or adjunctive regimen to DFO, is orally administered and can cross the blood-retina barrier. While degeneration of the RPE has been reported to occur under deferiprone [6, 7], newer studies have demonstrated that it can be a retinal protective iron chelator [15–17]. Finally, deferasirox is a newer oral efficient iron chelator without documented retinal penetration, while cases of reversible toxicity have also been reported. [18, 19]

The standard dose of DFO for chronic transfusional hemosiderosis in patients undergoing chronically repeated blood transfusions is 25–50 mg/kg/day, while a dose of 100 mg/kg/day or more is probable of toxicity [20]. It has been proposed that a daily dose of 50 mg/kg is the upper safe limit for ocular toxicity [21]. Moreover, DFO administered intravenously has been found to carry a higher risk of toxicity than the subcutaneous or intramuscular route [22].

Manifestations of DFO-induced retinal toxicity include RPE changes either at the posterior pole or at the periphery of the retina. Fundus autofluorescence examination in 197 patients with β-TM under DFO treatment, showed a 9% incidence of retinal abnormalities, which were classified, based on their severity, as minimal, focal, patchy and speckled [23]. Out of those, eyes with the patchy pattern showed further RPE damage as demonstrated with FAF, whereas patients with the focal and speckled patterns showed little or no change on follow up examinations with FAF. Visual acuity deteriorated in all eyes except from those with minimal FAF changes [23]. Pattern dystrophy-like lesions have also been described in patients undergoing prolonged DFO treatment (Table 1). Reviewing the cases in Table 1 we found 4 published cases of vitelliform maculopathy and 3 cases of butterfly shaped macular lesions associated with DFO in literature [24–26].

**Table 1:** Published pattern dystrophy cases associated with chelation therapy

Study (Year)	Study type No of Patients	DFO Treatment	Electrophysiology Tests	Retinal Findings	Additional information	Management	Follow up - outcome
Genead et al. [26]	Case report 1 Case	SQ N/A	ERG normal	Vitelliform Macular Lesion	Splenectomy Mild hearing impairment	N/A regarding chelation therapy Brinzolamide 0.1% Initiation	Improvement of vitelliform lesion in one eye with Brinzolamide 0.1% drops
Gonzales et al. [25]	Case series 2 Patients	PT1 1 g/2xdaily/SQ for 11 months, PT2 IV 2680 mg/5x weekly for 3 years	PT1 ERG, EOG normal PT2 ERG reduction in cone mediated responses EOG normal	Vitelliform Macular Lesion	Myelodysplasia	PT1 initially DFO dose reduced to 500 mg/day SQ, at 4 months increased to 1 g/d, discontinued at 1 year PT2 DFO discontinuation	PT1 Vision deteriorated, vitelliform lesion increased and atrophy developed at 2 years follow up, inspite of DFO dose modification and final discontinuation PT2 Vision deteriorated at 1 year follow up Lesion anatomically unchanged in one eye, resolution of vitelliform lesion in the fellow eye
Viola et al. [24]	Retrospective 10/20 Chart Review Study 20/290	SQ Range 1.5–4.5 mg/kg Mean 32.7 ± 8.7 years Range 20–52 years	N/A	Butterfly shaped macular lesion [3] Vitelliform macular lesion [1] Fundus flavimaculatus-like [3] Fundus pulvirulentus-like [3] Minimal change [10]	Beta-thalassemia	Vitelliform PT switched to deferasirox 1 butterfly PT switched to deferasirox, 2 unchanged	Mean duration of follow up 19.7 ± 8.8 months (range 10–45 months). 6 patients (one with Butterfly shaped and one with Vitelliform-like macular lesion) switched to deferasirox 14 patients remained on the same chelation regimen All Butterfly shaped and Vitelliform cases progressed to RPE atrophy during follow up
Bui et al. [35]	Case report 1 Case	N/A 16 g/week for 5 years	ERG diffuse rod dysfunction in one eye diffuse dysfunction in EOG in both eyes	Vitelliform Macular Lesion	Myelodysplasia Adult onset hearing loss	DFO discontinuation Switch to deferasirox Brinzolamide 0.1% Initiation	Lesion reduced in size 2 months after DFO discontinuation then increased on deferasirox Vision remained stable PT lost on follow up

Abbreviations used: *DFO* deferoxamine, *SQ* subcutaneous, *ERG* electroretinogram, *EOG* electrooculogram, *PT* patient, *N/A* not available

The exact mechanism of DFO toxicity, although thoroughly studied, remains unclear. Rahi et al. noticed thickening of Bruch’s membrane and depigmentation and degeneration of the RPE cells in microscopic examination of eyes with DFO retinopathy [27]. Pathophysiology of retinal damage in patients chronically treated with DFO is supposed to occur due to disruption of iron homeostasis in the retina [28] as well as chelation of other metals vital for proper retinal function such as copper, cobalt, zinc and nickel. [29, 30] In addition, DFO has been found to exhibit a direct p38 mediated toxic effect to RPE cells in in vitro studies [31]. In respect to pattern dystrophies in patients receiving DFO, histologic studies are required to elucidate the pathogenetic process of fluorophore buildup between RPE and outer photoreceptor segments that lead to the development of such lesions.

Electrophysiology testing has been proposed as a means for monitoring retinal function in cases of suspected DFO toxicity. Electroretinogram and electrooculogram (EOG) are usually confirmatory of DFO retinopathy and appear to be more sensitive in detecting early retinal damage than fundoscopy alone [3]. A recent study suggests that ffERG and multi-focal ERG are more sensitive in early damage detection than visual evoked potential, FAF imaging and OCT scans in patients under chelation therapy [32]. For instance, there are reports of bilateral reductions in response densities at the central retina corresponding to RPE changes related to DFO toxicity [3, 33, 34]. Repeated examinations with multifocal ERG have also been used for the follow up of patients with DFO related maculopathy in order to record functional changes. However, due to lack of specificity of these tests for DFO toxicity, various different results have been presented in literature [33]. Regarding pattern dystrophies, various contradicting electrophysiology findings have been reported in literature (Table 1). More specifically, ERG results range from normal [3, 26] to impaired cone [25] or rod responses [35]. Diffuse dysfunction in EOG has also been reported in one vitelliform case [35]. In our cases, patients had normal cone and rod responses in the full field ERG.

Cessation of DFO treatment has been reported to reverse early DFO toxicity related changes [36]. Kertes et al. however, reported progressive deterioration in mfERG during the first 4 months after discontinuation of DFO, which did not stabilize until 8 months later [37]. Regarding macular pattern-like dystrophies, limited information exists in literature on the possible effect of DFO cessation or DFO dose modification (Table 1). Viola et al. reported switch of chelation treatment, from DFO to deferasirox, in a patient with a vitelliform-like lesion as well as one patient with butterfly shaped macular changes while chelation therapy remained unchanged on two patients with butterfly shaped-like macular changes [24]. Interestingly, progressive RPE atrophy was demonstrated during follow-up in all of the above four patients, although resolution of the hyperreflective material had initially been observed in the patient with the vitelliform-like lesion [24]. In the cases reported by Gonzales visual deterioration occurred despite DFO discontinuation [25]. Finally, administration of brinzolamide 0.1% in a patient with vitelliform maculopathy related to DFO resulted in reduction of the macular lesion [38]. In a recent case report of a pseudovitelliform maculopathy related to DFO, further deterioration occurred after changing the chelator from DFO to deferasirox [35].

In this report, we present two cases of relatively rare pattern dystrophy like macular changes in patients treated with DFO for a prolonged period of time. Multimodal imaging utilization in our patients allowed a better evaluation and possibly earlier detection of the DFO-related changes. The correlation between DFO and the retinal changes presented in our patients is enhanced by the absence of any family history of macular pathology, the chronic treatment with DFO and the symmetric and bilateral appearance of macular changes. The age of presentation of visual disturbances in both patients is the 5th and 6th decade of life respectively, which also favors the diagnosis of an adult onset macular dystrophy. However, exclusion of possible genetic defects would further support the conclusion of an acquired pattern dystrophy of the macula induced by desferrioxamine. Adjustment of chelation treatment could have been a more proactive approach. However, in our cases, haematologist consultation suggested that patients should remain on the prescribed chelation therapy and be closely followed-up every three months. No sign of deterioration was observed over a period of 2 and 4 years respectively. Screening is therefore important for early detection, prompt diagnosis and follow up of possible drug-related toxicity in this particular group of patients.

## Abbreviations

BCVA: best-corrected visual acuity
DFO: deferoxamine
EOG: electrooculogram
FAF: fundus autofluorescence
ffERG: full-field electroretinogram
IOP: intraocular pressure
OD: right eye
OS: left eye
OU: both eyes
PXE: pseudoxanthoma elasticum
RPE: retinal pigment epithelium
SD-OCT: Spectral-Domain Optical Coherence Tomography
β-TI: beta-thalassemia intermedia
β-TM: Beta-thalassemia major
